# 

**Published:** 1849-01

**Authors:** 


### NOTICE TO READERS AND CORRESPONDENTS.

Our exchanges will all please direct to us at Chicago for the present.

We thank our friends for the liberality with which they contribute to our pages.

Reports of cases, illustrating modes of treatment, drawn up in a plain and clear manner, and giving all the symptoms of the diseases, form the most welcome contributions.

We have received, since our last, articles from Drs. Hansford, Brackett, Sedgwick, Irwin, Banks, Cooper, and a roll of papers from the Union Medical Society of Northern Indiana, most of which will appear in due time.

Also, the Safety Mask, by Dr. Lewis, of Frankfort, Ky.

We have received by express from the publishers:—

Medical Lexicon of Modern Terminology; being a complete vocabulary of definitions, including all the technical terms employed by writers and teachers of medical science at the present day, and comprising several hundreds of words not found in any other dictionary. Designed for the use of students and practitioners. Second edition greatly enlarged. By D. Merideth Reese, M. D., L. L. D., resident physician of Belvue Hospital, N. Y., Editor of Cooper's Surgical Dictionary, &c. pp. 250, 12mo. New York, S. S. and W. Wood, 1848.

An introductory lecture, delivered to the class of Stariing Medical College, Nov. 1, 1848. By F. Merrick, A. M., M. D., Professor of Chemistry.

An introductory lecture, delivered before the class of the medical department of St. Louis University; session 1848-9. By Jas. Blake, M. D., Professor of special, general, and surgical Anatomy, &c.

A Plea for Obstetrics. Introductory lecture to the course of Midwifery, in the medical department of Pennsylvania College; session 1848-9. By Jno. Willbank, M. D. Published by the class.

Also in exchange:—

Buffalo Medical Journal for December.
New York Journal of Medicine for Nov.
Boston Medical and Surgical Journal, up to December 20.
Ohio Medical and Surgical Journal for Nov.
Southern Medical and Surgical Journal Dec.
Medical News and Library, December.
The Journal of Heath, and Miscellany, Nov.
Western Journal of Medicine and Surgery, December.
Western Lancet and Hospital Reporter Dec.
The Annalist for December 1.
New Orleans Medical and Surgical Journal November.
American Journal of Pharmacy November.
New Jersey Medical Reporter October.
Charleston Medical Journal and Review November.
St. Louis Medical and Surgical Journal November and December.
Medical Examiner for December.
American Journal of Insanity, October.

---

### RECEIPTS FOR THIS JOURNAL FOR NOVEMBER AND DECEMBER, 1848.

| | |
|---|---|
| **ILLINOIS.** | |
| Drs. W. W. Welch, - - - - | $2 00 |
| H. T. Brown, - - - - | 2 00 |
| R. R. Stone, - - - - | 1 00 |
| F. Scammon, - - - - | 2 00 |
| W. F. Coleman, - - - - | 2 00 |
| W. W. Sedgwick, - - - - | 2 00 |
| J. Pearson - - - - | 2 00 |
| D. Hess - - - - - | 4 00 |
| A. W. Benton - - - - | 2 00 |
| **INDIANA.** | |
| Drs. C. C. Garrett, - - - - | $2 00 |
| Jno. Lewis, - - - - | 2 00 |
| Geo. Manners, - - - - | 2 00 |
| W. Dickey, - - - - | 2 00 |
| Drs. I. Landers, - - - - | 4 00 |
| G.M. Huggans, - - - - | 2 00 |
| M. W. Gentry, - - - - | 2 00 |
| **MICHIGAN.** | |
| Drs. Jno. McLean, - - - - | $2 00 |
| S. Stoddard, - - - - | 2 00 |
| A. B. Crawford, - - - - | 2 10 |
| Jno. Acres, - - - - | 2 00 |
| W. H. Elliot, - - - - | 5 00 |
| C. W. Hall, - - - - | 1 00 |
| **WISCONSIN.** | |
| Drs. J. Campbell, - - - - | $2 00 |
| Clark and Rice, - - - - | 2 00 |
| **MISSOURI.** | |
| Dr. S. S. Platt, - - - - | $2 00 |

### NORTH-WESTERN
### MEDICAL AND SURGICAL JOURNAL.

EDITED BY

W. B. HERRICK, M. D., AND JOHN EVANS, M. D.

PROFESSORS IN RUSH MEDICAL COLLEGE.

THE NORTH-WESTERN MEDICAL AND SURGICAL JOURNAL

Is issued simultaneously at Chicago, Illinois, and Indianapolis, Indiana, on the first of every other month, beginning with May, in numbers, each containing from 88 to 96 pages, neatly printed with new type and on good paper, and making, at the end of the year, a volume of about 550 pages, octavo, with title page and index complete.

Contributions to our pages, especially from physicians of the North-west, are earnestly solicited.

TERMS.—Two Dollars per annum, *in advance*, or Eight Dollars in one remittance for five copies, which may be sent by mail at our risk or paid to either of the Editors or authorized agents.

CHICAGO:
PRINTED BY J. W. DUZAN & Co.,
*Corner of Clark and Randolph Streets.*

